# *catena*-Poly[[(5,5′-dimethyl- 2,2′-bipyridine-κ^2^ *N*,*N*′)cadmium(II)]-di-μ-chlorido]

**DOI:** 10.1107/S1600536808027657

**Published:** 2008-09-06

**Authors:** Roya Ahmadi, Aida Khalighi, Khadijeh Kalateh, Vahid Amani, Hamid Reza Khavasi

**Affiliations:** a Islamic Azad University, Shahr-e-Rey Branch, Tehran, Iran; b Department of Chemistry, Shahid Beheshti University, Tehran 1983963113, Iran

## Abstract

The asymmetric unit of the title compound, [CdCl_2_(C_12_H_12_N_2_)]_*n*_, contains one half-mol­ecule; a twofold rotation axis passes through the Cd atom. The Cd^II^ atom is six-coordinated in a distorted octa­hedral configuration by two N atoms from 2,2′-bipyridine-5,5′-dimethyl and four bridging Cl atoms. The bridging function of the chloro atoms leads to a one-dimensional chain structure. There is a π–π contact between the pyridine rings [centroid–centroid distance = 3.9807 (9) Å].

## Related literature

For related literature, see: Chen *et al.* (2003 ▶); Flook *et al.* (1973 ▶); Hu & Englert (2002 ▶); Janiak *et al.* (1999 ▶); Satoh *et al.* (2001 ▶); Zhou *et al.* (2003 ▶); Khalighi *et al.* (2008 ▶).
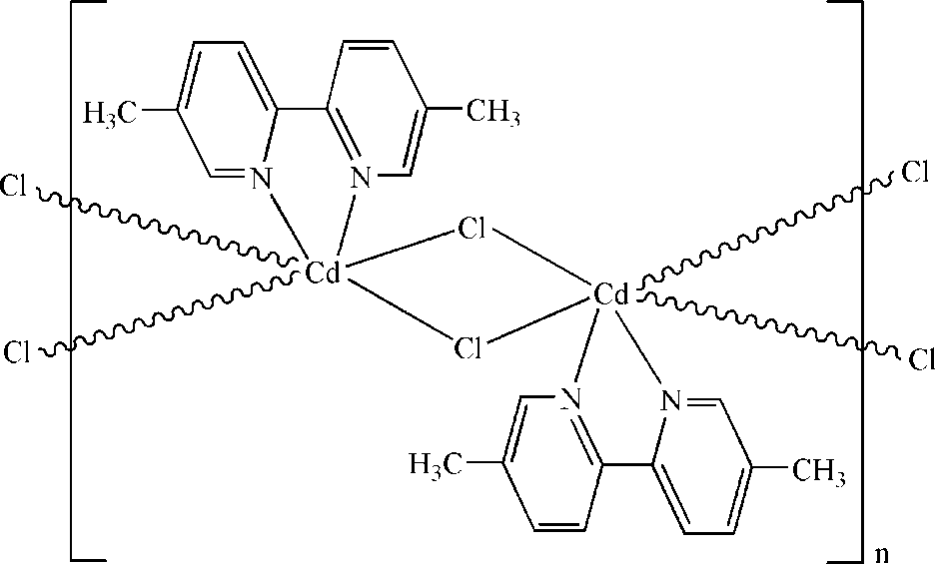


## Experimental

### 

#### Crystal data

[CdCl_2_(C_12_H_12_N_2_)]
*M* *_r_* = 367.55
Monoclinic, 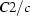

*a* = 20.365 (4) Å
*b* = 9.3135 (19) Å
*c* = 7.2313 (14) Å
β = 107.53 (3)°
*V* = 1307.9 (5) Å^3^
*Z* = 4
Mo *K*α radiation
μ = 2.06 mm^−1^
*T* = 298 (2) K
0.20 × 0.17 × 0.15 mm

#### Data collection

Bruker SMART CCD area-detector diffractometer
Absorption correction: multi-scan (*SADABS*; Sheldrick, 1998 ▶) *T*_min_ = 0.666, *T*_max_ = 0.740
4283 measured reflections
1724 independent reflections
1585 reflections with *I* > 2σ(*I*)
*R*_int_ = 0.052

#### Refinement

*R*[*F* ^2^ > 2σ(*F* ^2^)] = 0.032
*wR*(*F* ^2^) = 0.089
*S* = 1.08
1724 reflections
78 parameters
H-atom parameters constrained
Δρ_max_ = 0.68 e Å^−3^
Δρ_min_ = −0.76 e Å^−3^

### 

Data collection: *SMART* (Bruker, 1998 ▶); cell refinement: *SAINT* (Bruker, 1998 ▶); data reduction: *SAINT*; program(s) used to solve structure: *SHELXTL* (Sheldrick, 2008 ▶); program(s) used to refine structure: *SHELXTL*; molecular graphics: *ORTEP-3 for Windows* (Farrugia, 1997 ▶); software used to prepare material for publication: *WinGX* (Farrugia, 1999 ▶).

## Supplementary Material

Crystal structure: contains datablocks I. DOI: 10.1107/S1600536808027657/hk2520sup1.cif

Structure factors: contains datablocks I. DOI: 10.1107/S1600536808027657/hk2520Isup2.hkl

Additional supplementary materials:  crystallographic information; 3D view; checkCIF report

## Figures and Tables

**Table:** 

Cd1—Cl1^i^	2.7668 (10)
Cl1—Cd1	2.5457 (9)
N1—Cd1	2.355 (2)

**Table:** 

Cl1—Cd1—Cl1^i^	85.18 (2)
Cl1—Cd1—Cl1^ii^	96.22 (3)
Cl1^i^—Cd1—Cl1^ii^	177.73 (2)
Cl1^iii^—Cd1—Cl1	104.77 (4)
N1—Cd1—Cl1^iii^	159.71 (6)
N1—Cd1—Cl1	93.57 (6)
N1^iii^—Cd1—Cl1	159.71 (6)
N1—Cd1—Cl1^i^	93.89 (5)
N1—Cd1—Cl1^ii^	84.24 (5)
N1—Cd1—N1^iii^	69.98 (10)

Symmetry codes: (i) 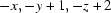; (ii) 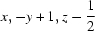; (iii) 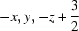.

**Fig. 1.**
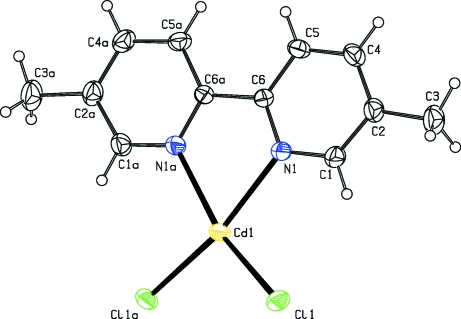
The molecular structure of the title molecule, with the atom-numbering scheme. Displacement ellipsoids are drawn at the 50% probability level [symmetry code: (a) -x, y, 3/2 - z].

**Fig. 2.**
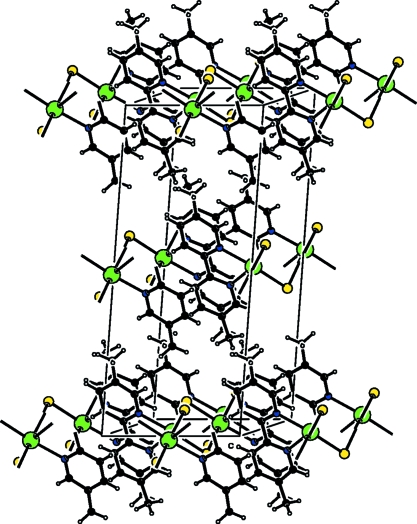
A packing diagram of the title compound.

## supplementary crystallographic information

### Comment

In a recent paper, we reported the synthesis and crystal structure of [Zn(5,5'-dmbpy)Cl_2_], (Khalighi *et al.*, 2008) [where 5,5'-dmbpy is 5,5'-dimethyl-2,2'-bipyridine]. Several Cd^II^ polymer complexes, with formula, [Cd(N—N)(µ-Cl)_2_]_n_, such as [Cd(phen)(µ-Cl)_2_]_n_, (II) (Chen *et al.*, 2003), {[Cd(5,5'-dabpy)(µ-Cl)_2_].2H_2_O}_n_, (III) (Janiak *et al.*, 1999) and [Cd(bipy)(µ-Cl)_2_]_n_, (IV) (Zhou *et al.*, 2003) [where bipy is 2,2'-bipyridine, 5,5'-dabpy is 5,5'-diamino -2,2'-bipyridine and phen is 1,10-phenanthroline] have been synthesized and characterized by single-crystal X-ray diffraction methods. There are also several Cd^II^ polymer complexes, with formula, [Cd(µ-Cl)_2_*L*_2_]_n_, such as [Cd(µ-Cl)_2_(3,5-Me_2_py)_2_]_n_, (V), [Cd(µ-Cl)_2_(3,5-Br_2_py)_2_]_n_, (VI) and [Cd(µ-Cl)_2_(3,5-Cl_2_py)_2_]_n_, (VII) (Hu & Englert, 2002), [Cd(µ-Cl)_2_(3-Mepy)_2_]_n_, (VIII) (Satoh, *et al.*, 2001) and [Cd(µ-Cl)_2_(im)_2_]_n_, (IX) (Flook *et al.*, 1973) [where py is pyridine and im is imidazole] have been synthesized and characterized by single-crystal X-ray diffraction methods. We report herein the synthesis and crystal structure of the title compound (I).

The asymmetric unit of the title compound, (I), contains one half-molecule (Fig. 1). The Cd^II^ atom is six-coordinated in a distorted octahedral configuration by two N atoms from 2,2'-bipyridine-5,5'-dimethyl and four bridging Cl atoms. The bridging function of chloro atoms leads to a one-dimensional chain structure. The Cd—Cl and Cd—N bond lengths and angles (Table 1) are within normal ranges, as in (II), (III) and (IV).

In the crystal structure, the π—π contact (Fig. 2) between the pyridine rings, Cg4···Cg4^i^ [symmetry code: (i) x, 1/2- y, z, where Cg4 is centroid of the ring (N1/C1/C2/C4-C6)] may stabilize the structure, with centroid-centroid distance of 3.9807 (9) Å.

### Experimental

For the preparation of the title compound, a solution of 5,5'-dimethyl-2,2' -bipyridine (0.25 g, 1.33 mmol) in methanol (10 ml) was added to a solution of CdCl_2_.H_2_O (0.27 g, 1.33 mmol) in methanol (10 ml) at room temperature. The suitable crystals for X-ray analysis were obtained by methanol diffusion to a colorless solution in DMSO. Suitable crystals were isolated after one week (yield; 0.35 g, 71.6%, m.p. < 573 K).

### Refinement

H atoms were positioned geometrically, with C-H = 0.93 and 0.96 Å for aromatic and methyl H, respectively, and constrained to ride on their parent atoms with U_iso_(H) = 1.2U_eq_(C).

### Crystal data

**Table:**

[CdCl_2_(C_12_H_12_N_2_)]	*F*(000) = 720
*M**_r_* = 367.55	*D*_x_ = 1.867 Mg m^−^^3^
Monoclinic, *C*2/*c*	Mo *K*α radiation, λ = 0.71073 Å
Hall symbol: -C 2yc	Cell parameters from 1004 reflections
*a* = 20.365 (4) Å	θ = 4.1–29.2°
*b* = 9.3135 (19) Å	µ = 2.06 mm^−^^1^
*c* = 7.2313 (14) Å	*T* = 298 K
β = 107.53 (3)°	Block, colorless
*V* = 1307.9 (5) Å^3^	0.20 × 0.17 × 0.15 mm
*Z* = 4	

### Data collection

**Table:**

Bruker SMART CCD area-detector diffractometer	1724 independent reflections
Radiation source: fine-focus sealed tube	1585 reflections with *I* > 2σ(*I*)
graphite	*R*_int_ = 0.052
φ and ω scans	θ_max_ = 29.2°, θ_min_ = 4.1°
Absorption correction: multi-scan (SADABS; Sheldrick, 1998)	*h* = −27→18
*T*_min_ = 0.666, *T*_max_ = 0.740	*k* = −12→11
4283 measured reflections	*l* = −9→9

### Refinement

**Table:**

Refinement on *F*^2^	Primary atom site location: structure-invariant direct methods
Least-squares matrix: full	Secondary atom site location: difference Fourier map
*R*[*F*^2^ > 2σ(*F*^2^)] = 0.032	Hydrogen site location: inferred from neighbouring sites
*wR*(*F*^2^) = 0.089	H-atom parameters constrained
*S* = 1.08	*w* = 1/[σ^2^(*F*_o_^2^) + (0.0543*P*)^2^ + 0.9451*P*] where *P* = (*F*_o_^2^ + 2*F*_c_^2^)/3
1724 reflections	(Δ/σ)_max_ = 0.011
78 parameters	Δρ_max_ = 0.68 e Å^−^^3^
0 restraints	Δρ_min_ = −0.76 e Å^−^^3^

### Special details

**Table:**

Geometry. All e.s.d.'s (except the e.s.d. in the dihedral angle between two l.s. planes) are estimated using the full covariance matrix. The cell e.s.d.'s are taken into account individually in the estimation of e.s.d.'s in distances, angles and torsion angles; correlations between e.s.d.'s in cell parameters are only used when they are defined by crystal symmetry. An approximate (isotropic) treatment of cell e.s.d.'s is used for estimating e.s.d.'s involving l.s. planes.
Refinement. Refinement of *F*^2^ against ALL reflections. The weighted *R*-factor *wR* and goodness of fit *S* are based on *F*^2^, conventional *R*-factors *R* are based on *F*, with *F* set to zero for negative *F*^2^. The threshold expression of *F*^2^ > σ(*F*^2^) is used only for calculating *R*-factors(gt) *etc*. and is not relevant to the choice of reflections for refinement. *R*-factors based on *F*^2^ are statistically about twice as large as those based on *F*, and *R*- factors based on ALL data will be even larger.

### Fractional atomic coordinates and isotropic or equivalent isotropic displacement parameters (Å^2^)

**Table:**

	*x*	*y*	*z*	*U*_iso_*/*U*_eq_	
Cd1	0.0000	0.58047 (2)	0.7500	0.03912 (12)	
Cl1	0.07886 (4)	0.41364 (6)	0.99832 (11)	0.04502 (17)	
N1	0.06292 (10)	0.7876 (2)	0.8828 (3)	0.0383 (4)	
C1	0.12723 (13)	0.7815 (3)	1.0067 (4)	0.0460 (5)	
H1	0.1458	0.6918	1.0488	0.055*	
C2	0.16716 (14)	0.9023 (3)	1.0746 (5)	0.0480 (6)	
C3	0.23917 (17)	0.8878 (5)	1.2093 (6)	0.0674 (9)	
H3A	0.2665	0.8339	1.1466	0.081*	
H3B	0.2381	0.8389	1.3251	0.081*	
H3C	0.2589	0.9815	1.2422	0.081*	
C4	0.13695 (15)	1.0347 (3)	1.0145 (4)	0.0484 (6)	
H4	0.1612	1.1187	1.0594	0.058*	
C5	0.07094 (15)	1.0418 (3)	0.8883 (4)	0.0435 (5)	
H5	0.0504	1.1303	0.8486	0.052*	
C6	0.03548 (12)	0.9157 (2)	0.8213 (4)	0.0344 (4)	

### Atomic displacement parameters (Å^2^)

**Table:**

	*U*^11^	*U*^22^	*U*^33^	*U*^12^	*U*^13^	*U*^23^
Cd1	0.04181 (17)	0.02643 (15)	0.04102 (17)	0.000	0.00027 (11)	0.000
Cl1	0.0458 (3)	0.0370 (3)	0.0479 (3)	0.0092 (2)	0.0074 (3)	0.0073 (2)
N1	0.0373 (9)	0.0313 (9)	0.0426 (10)	0.0009 (8)	0.0064 (8)	−0.0023 (8)
C1	0.0381 (11)	0.0426 (13)	0.0501 (13)	0.0045 (10)	0.0024 (10)	−0.0068 (11)
C2	0.0364 (12)	0.0536 (16)	0.0507 (14)	−0.0035 (10)	0.0080 (11)	−0.0133 (11)
C3	0.0394 (14)	0.082 (2)	0.070 (2)	−0.0029 (15)	−0.0003 (14)	−0.0177 (18)
C4	0.0448 (13)	0.0457 (14)	0.0529 (15)	−0.0112 (11)	0.0117 (11)	−0.0135 (12)
C5	0.0487 (13)	0.0303 (10)	0.0533 (14)	−0.0046 (10)	0.0179 (12)	−0.0062 (10)
C6	0.0339 (10)	0.0296 (11)	0.0406 (11)	0.0012 (7)	0.0129 (9)	−0.0020 (8)

### Geometric parameters (Å, °)

**Table:**

Cd1—Cl1^i^	2.5457 (9)	C2—C3	1.502 (4)
Cd1—Cl1^ii^	2.7668 (10)	C3—H3A	0.9600
Cd1—Cl1^iii^	2.7668 (10)	C3—H3B	0.9600
Cl1—Cd1	2.5457 (9)	C3—H3C	0.9600
Cl1—Cd1^ii^	2.7668 (10)	C4—C5	1.380 (4)
Cd1—N1^i^	2.355 (2)	C4—H4	0.9300
N1—Cd1	2.355 (2)	C5—C6	1.387 (3)
C1—N1	1.347 (3)	C5—H5	0.9300
C1—C2	1.389 (4)	C6—N1	1.336 (3)
C1—H1	0.9300	C6—C6^i^	1.501 (5)
C2—C4	1.388 (4)		
			
Cd1—Cl1—Cd1^ii^	94.82 (2)	N1—C1—H1	118.3
Cl1^i^—Cd1—Cl1^ii^	96.22 (3)	C2—C1—H1	118.3
Cl1—Cd1—Cl1^ii^	85.18 (2)	C4—C2—C1	116.9 (3)
Cl1^i^—Cd1—Cl1^iii^	85.18 (2)	C4—C2—C3	122.5 (3)
Cl1—Cd1—Cl1^iii^	96.22 (3)	C1—C2—C3	120.6 (3)
Cl1^ii^—Cd1—Cl1^iii^	177.73 (2)	C2—C3—H3A	109.5
Cl1^i^—Cd1—Cl1	104.77 (4)	C2—C3—H3B	109.5
N1—Cd1—Cl1^i^	159.71 (6)	H3A—C3—H3B	109.5
N1^i^—Cd1—Cl1^i^	93.57 (6)	C2—C3—H3C	109.5
N1—Cd1—Cl1	93.57 (6)	H3A—C3—H3C	109.5
N1^i^—Cd1—Cl1	159.71 (6)	H3B—C3—H3C	109.5
N1—Cd1—Cl1^ii^	93.89 (5)	C5—C4—C2	120.1 (3)
N1^i^—Cd1—Cl1^ii^	84.24 (5)	C5—C4—H4	120.0
N1—Cd1—Cl1^iii^	84.24 (5)	C2—C4—H4	120.0
N1^i^—Cd1—Cl1^iii^	93.89 (5)	C4—C5—C6	119.4 (3)
N1—Cd1—N1^i^	69.98 (10)	C4—C5—H5	120.3
C6—N1—C1	119.0 (2)	C6—C5—H5	120.3
C6—N1—Cd1	118.31 (15)	N1—C6—C5	121.2 (2)
C1—N1—Cd1	122.49 (18)	N1—C6—C6^i^	116.64 (13)
N1—C1—C2	123.3 (3)	C5—C6—C6^i^	122.15 (16)
			
N1—C1—C2—C4	2.3 (5)	C1—N1—Cd1—N1^i^	−176.1 (3)
N1—C1—C2—C3	−178.6 (3)	C6—N1—Cd1—Cl1^i^	36.2 (3)
C1—C2—C4—C5	−1.9 (4)	C1—N1—Cd1—Cl1^i^	−138.71 (19)
C3—C2—C4—C5	179.1 (3)	C6—N1—Cd1—Cl1	−168.97 (17)
C2—C4—C5—C6	−0.5 (4)	C1—N1—Cd1—Cl1	16.1 (2)
C4—C5—C6—N1	2.8 (4)	C6—N1—Cd1—Cl1^ii^	−83.57 (18)
C4—C5—C6—C6^i^	−177.9 (3)	C1—N1—Cd1—Cl1^ii^	101.5 (2)
C5—C6—N1—C1	−2.5 (4)	C6—N1—Cd1—Cl1^iii^	95.14 (18)
C6^i^—C6—N1—C1	178.2 (3)	C1—N1—Cd1—Cl1^iii^	−79.8 (2)
C5—C6—N1—Cd1	−177.59 (18)	Cd1^ii^—Cl1—Cd1—N1	93.61 (5)
C6^i^—C6—N1—Cd1	3.0 (3)	Cd1^ii^—Cl1—Cd1—N1^i^	58.77 (15)
C2—C1—N1—C6	−0.1 (4)	Cd1^ii^—Cl1—Cd1—Cl1^i^	−95.17 (2)
C2—C1—N1—Cd1	174.8 (2)	Cd1^ii^—Cl1—Cd1—Cl1^ii^	0.0
C6—N1—Cd1—N1^i^	−1.14 (13)	Cd1^ii^—Cl1—Cd1—Cl1^iii^	178.20 (2)

Symmetry codes: (i) −*x*, *y*, −*z*+3/2; (ii) −*x*, −*y*+1, −*z*+2; (iii) *x*, −*y*+1, *z*−1/2.
